# Evaluation of carotid arterial elasticity in patients with obstructive sleep apnea hypopnea syndrome by two-dimensional speckle tracking imaging

**DOI:** 10.1097/MD.0000000000008817

**Published:** 2017-12-22

**Authors:** Manjie You, Li Zhang, Lingyun Fang, Jie Li, Mingxing Xie

**Affiliations:** a Department of Ultrasound, Union Hospital, Tongji Medical College, Huazhong University of Science and Technology; b Hubei Province Key Lab of Molecular Imaging, Wuhan, China.

**Keywords:** carotid artery, sleep apnea, speckle tracking, stiffness

## Abstract

The objective is to evaluate carotid arterial elasticity in patients with obstructive sleep apnea hypopnea syndrome (OSAHS) by two-dimensional speckle tracking imaging (2D-STI).

Sixty-two hospitalized patients with OSAHS and 20 healthy subjects were studied. The OSAHS patients were divided into 3 subgroups: a mild group, a moderate group, and severe group. All subjects underwent complete left common carotid artery (LCCA) examination by echo-tacking technique and 2D-STI. The stiffness parameter (β), elastic modulus (Eρ), stiffness β single pulse wave velocity (PWVβ), and arterial compliance (AC) were automatically calculated by echo-tracking technique. And the global and segmental peak systolic circumferential artery strain (CAS) values were made off-line using 2D-STI.

The β, Ep, and PWVβ values of the carotid artery in the moderate and severe groups were greater than those in the control group (*P* < .05). In addition, the systolic peak global CAS and the segments between 5 and 7 o’clock in the moderate and severe groups were lower than those in the control group (*P* < .05). Compared with mild group, the β, PWVp, and Ep values of the carotid artery in the moderate and severe groups were higher (*P* < .05) and the systolic peak global CAS lower than in the control group (*P* < .05). The systolic peak global CAS was significantly inversely correlated with stiffness (β, r = − 0.61, *P* < .05) and stiffness β single pulse wave velocity (PWVβ, r = −0.59, *P* < .05). Through stepwise multiple linear regression analysis, age and SaO2 were the significant variables that determined the systolic peak global CAS

2D-STI provides a new method to investigate carotid arterial elasticity in patients with OSAHS.

## 1 Introduction

Obstructive sleep apnea hypopnea syndrome (OSAHS) is associated with sympathetic hyperactivity, excessive nocturnal sweating, sleepiness, and neuro behavioral cognitive alterations and is closely related to the occurrence of cardiovascular system diseases. Long-term anoxic conditions cause pathological changes to the cardiovascular systems of OSAHS patients. Hypertension (HT) is an independent risk factor for OSAHS, and approximately 50% to 60% of OSAHS patients have HT. OSAHS is closely associated with other cardiovascular diseases, including 20% to 30% of combined coronary artery disease, 20% to 30% of merged pulmonary hypertension, and 5% to 10% of congestive heart failure.^[1]^ At present, little research concerning the mechanics of carotid function and structure in OSAHS patients is available. Therefore, the purpose of this study was to evaluate carotid arterial elasticity in patients with OSAHS by two-dimensional speckle tracking imaging (2D-STI). Carotid artery systolic peak global circumferential artery strain (CAS) and vascular elasticity parameters from echo-tracking technique were also evaluated.

## 2 Methods

### 2.1 Study population

From May 2014 to December 2014, 62 consecutive OSAHS patients (52 males, 10 females, mean age 42.3 ± 11.2 years, range 31.1–52.9 years) were enrolled in this study. They were divided into 3 subgroups according to the apnea-hypopnea index (AHI) and arterial oxygen saturation (SaO2): the mild group (AHI: 5–20, SaO2>86%, n = 17), the moderate group (AHI: 21–50, SaO2: 80%–85%, n = 20), and the severe group (AHI>51, SaO2<86%, n = 25).

Inclusion criteria were as follows: matched with 2012 American academy of sleep medicine (AASM) manual for the scoring of sleep and associated events^[2]^ diagnostic criteria of OSAHS, and undergo polysomnography included AHI > 5; the presence of 15 or more obstructive respiratory events per hour of sleep-related symptoms is also sufficient for the diagnosis of OSAHS due to the greater association of this severity of obstruction with important consequences such as increased cardiovascular disease (CVD) risk.^[2,3]^ The exclusive criteria were as follows:^[4,5]^ primary snoring, upper airway resistance syndrome (UARS), narcolepsy, restless legs syndrome.

The normal control group consisted of 20 age-matched healthy volunteers, including 17 males and 3 females with a mean age of 38.9 ± 8.2 years, range 31to 45 years old. They had no history of cardiovascular disease with sinus rhythm. Other organic diseases were excluded by physical examination, biochemical tests, electrocardiogram (ECG), and echo examination. Demographic characteristics and clinical date, including age, gender, weight, height, systolic blood pressure (SBP), and diastolic blood pressure (DBP) measurements, were collected for all subjects.

The study was approved by the institutional research ethics committee at Union Hospital, Tongji Medical College, Huazhong University of Science and Technology, China. All data used were anonymized. The subjects gave written informed consent. All procedures and data analysis were performed by the authors.

### 2.2 The echo-tracking technique

Patient blood pressure was measured 5 minutes after supine rest during the ECG recording. After conventional bilateral carotid artery scanning, the patient's neck was fully exposed by turning to the other side.

A commercially available ultrasound machine (α10, Japan Aloka Prosound) equipped with a frequency of 7.5 to13.0 MHz high-resolution vascular probe (Aloka Co Ltd, Tokyo Japan UST-5412) was used for all of the ultrasound examinations. The echo-tracking test position was under the margin of 1.5 cm from the carotid artery ball, and the anterior and posterior walls in the longitudinal section of the artery intima-media were shown most clearly when the sampling gate was placed at the junction of them idle and outer membranes. The M sampling line was regulated such that the vertical and carotid artery walls yielded the best images with the largest vessel diameters. Internal diastolic diameter (Dd) was the left common carotid artery (LCCA) dimension at end-diastole and systolic diameter (Ds) was LCCA dimension at end-systole.

**Figure 1:**
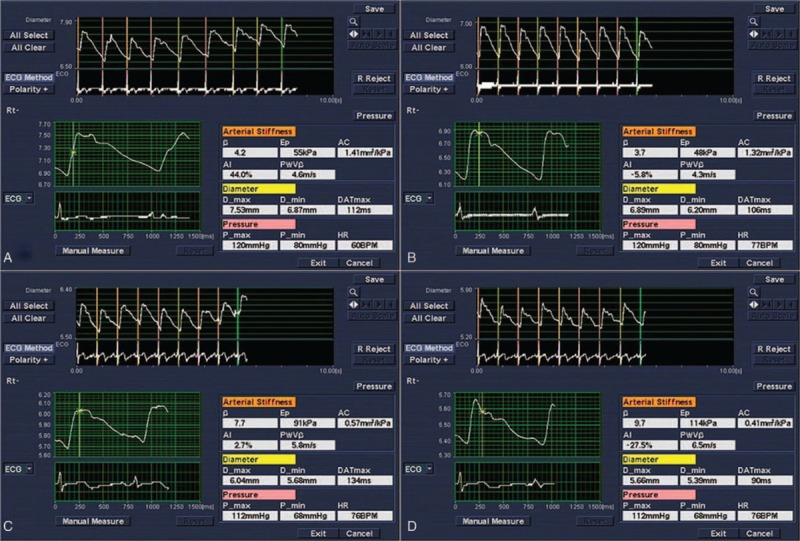
The measurement of carotid artery elasticity parameters by E-tracking technology. A, The control group. B, The mild group. C, The moderate group. D, The severe group.

The mean intima-media thickness (IMT) and maximal IMT of the far wall of the LCCA was measured using commercially available semiautomated edge-detection software (IMT Option, General Electric Medical System, Milwaukee, WI). The region of interest was placed from the beginning of carotid bulbs to a 2 cm proximal site in the LCCA. The echo-tracking technique was then initiated, and 7 or more cardiac cycle images were selected and input into the system. The computer could automatically calculate the appropriate parameters, including: stiffness (β), single pulse wave velocity (PWVβ), elastic modulus (Eρ), arterial compliance (AC), and augmentation index (AI) (Fig. 1).

### 2.3 2D-STI

**Figure 2:**
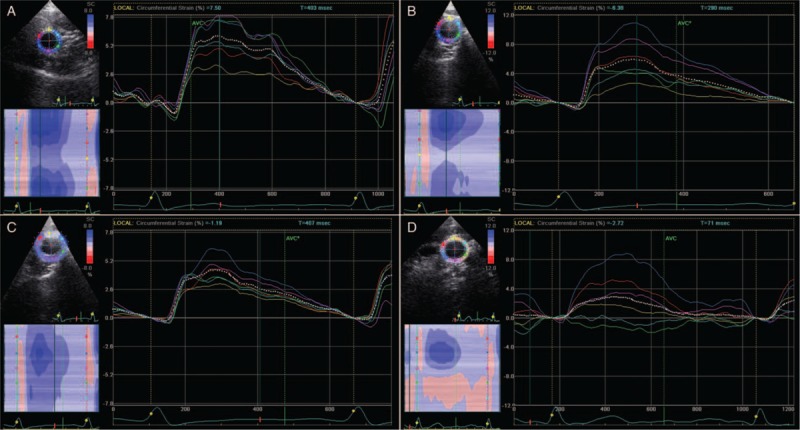
A representative circumferential carotid artery strain (CAS) curve of left common carotid artery. A, The control group. B, The mild group. C, The moderate group. D, The severe group.

Ultrasound images were acquired using a commercially available ultrasound system (Vivid 7; General Electric Medical Systems, Milwaukee, WI) equipped with a sector array probe (12 MHz, 10S; General Electric Medical Systems) for LCCA. Optimal short axis views of the LCCA were collected 1.5 cm below the bifurcation and divided into 6 regions (11 and 1 o’clock, 1 and 3 o’clock, 3 and 5 o’clock, 5 and 7 o’clock, 7 and 9 o’clock, and 9 and 11 o’clock). To obtain the optimal image for 2D-STI analysis, 2D image acquisition was performed at a frame rate of more than 70 to 170 frames per second, and 3 cardiac cycles were stored in cine-loop format for subsequent analyses. The LCCA was examined with the head tilted slightly upward in the midline portion. The transducer was manipulated such that the near and far walls of the LCCA were parallel to the transducer footprint, and the lumen diameter was maximized in the longitudinal plane. The LCCA diameter was measured at the end diastole. All 2D-STI strain measurements were averaged for at least 3 consecutive beats. The superficial probe pressure may affect the probe blood vessels to the adjacent segment and may be due to the heterogeneity of the ultrasonic linear density. The echo loss may result in unreliable strain measurement. Therefore, we selected the far field arterial wall section for analyzed (Fig. 2).

### 2.4 Statistical analysis

The values are expressed as the mean ± standard deviation. Analysis of covariance was used to compare CAS and β in subjects. Correlations between CAS, β and age were analyzed using the Pearson correlation analysis. Multivariate regression analysis that included all subjects was performed to determine the independent predictors of CAS. Intra- and interobserver agreement was calculated using the Bland–Altman method. *P* value less than .05 considered statistically significant. All statistical analyses were performed using statistical software (Statview version 5.0; SAS, Cary, NC).

### 2.5 Reproducibility

Interobserver and intraobserver variability of the measurements were assessed in 15 randomly selected subjects. To assess intraobserver variability, the same observer (MY) measured the left side of the LCCA CAS, and β values twice at an interval of 2 months to avoid recall bias. To assess interobserver variability, the left side of the LCCA CAS, and β values were performed by a second observer (JL), who was blinded to the results of the first observer (MY).

## 3 Results

There were no significant differences between the 4 groups regarding age, sex, height, body mass index, SBP, and DBP (*P* > .05) (Table 1).

**Table 1:**
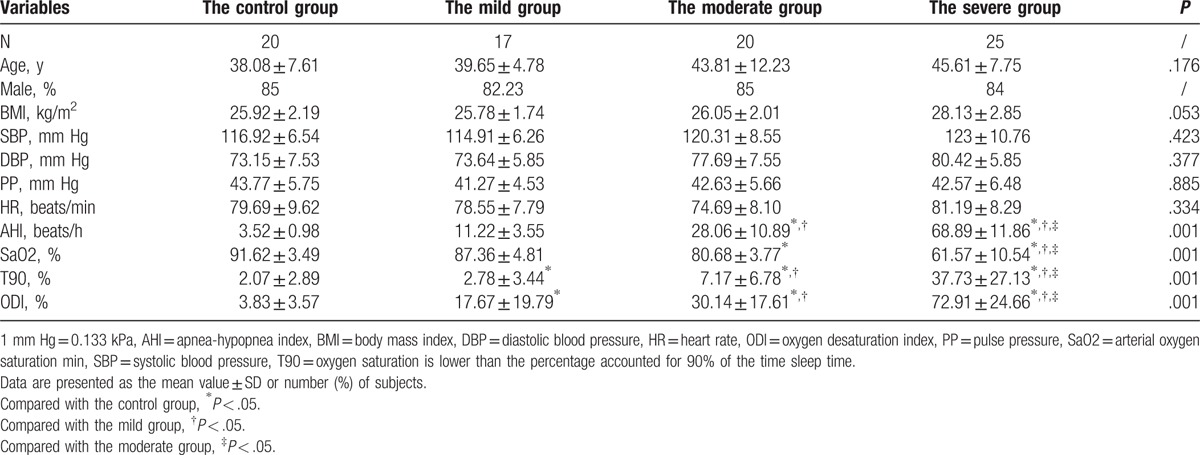
Clinical characteristics of the study subjects.

β, PWVβ, and Ep were increased in patients with OSAHS, while AC was lower significantly (*P* < .01), the values worsen in the severe group. The incidence of plaque was not significantly different between the 4 groups (*P*>.05). Compared with the control group, the Dd, β, Ep, and PWVβ values of the carotid artery in the moderate and severe groups were higher (*P* < .05), but the AC value was lower. There were no significant differences between the mild group and the control group regarding the Ds, Dd, β, Ep, PWVβ, and AC values (*P* > .05). Compared with mild group, the β, PWVβ, and Ep of carotid artery in moderate and severe groups were higher (*P* < .05), but the AC were lower (*P* < .05). There were no significant differences among the mild, moderate, severe groups, and the control group regarding the IMT (*P* > .05) (Table 2, Fig. 1).

**Table 2:**
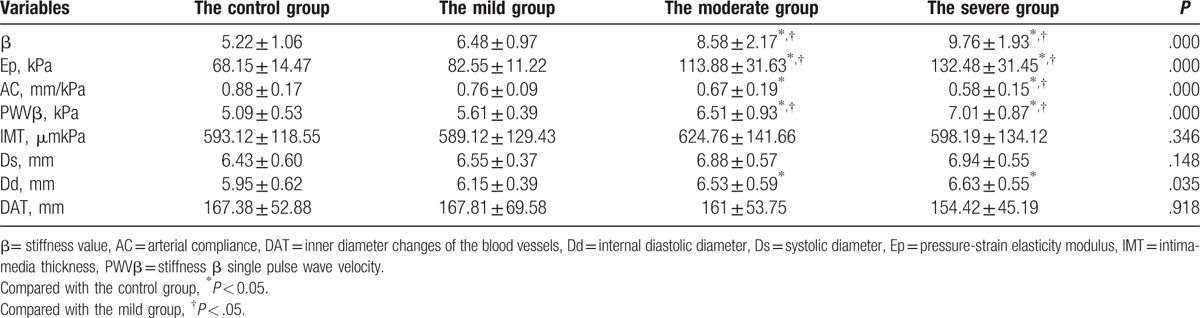
The carotid artery stiffness parameters.

The systolic peak global CAS was lower significantly in patients with OSAHS (*P* < .01), the values worsen in the severe group. The incidence of plaque was not significantly different between the 4 groups (*P*>.05). Compared with the control group, the systolic peak global CAS and the segments of between 5 and 7 o’clock in the moderate and severe groups were lower (*P* < .05). Compared with mild group, the systolic peak global CAS in the moderate and severe groups was lower (*P* < .05). There were no significant differences between the mild group and the control group in the systolic peak global CAS and the segments between 3 and 5 o’clock, 5 and 7 o’clock, and 7 and 9 o’clock (Table 3, Fig. 2).

**Table 3:**
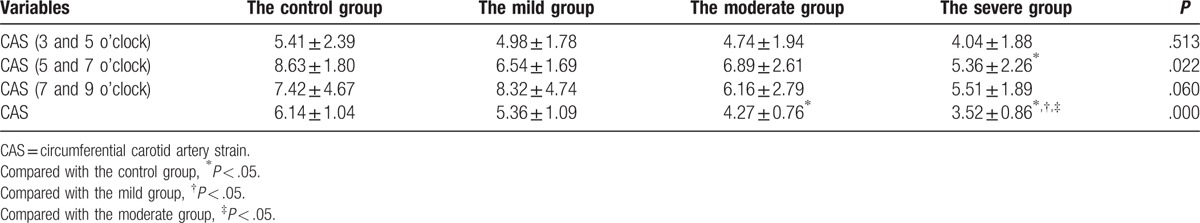
The carotid artery circumferential strain.

**Figure 3:**
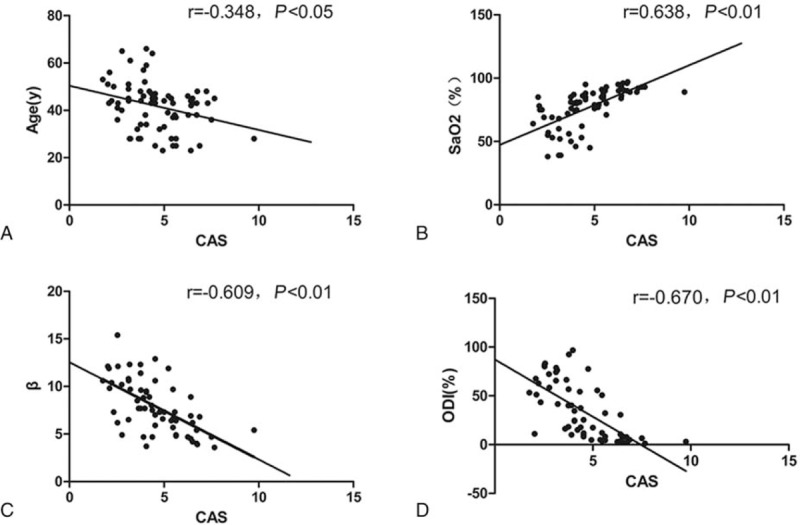
The correlationship of between CAS, ODI, SaO2, and β and age. β = stiffness parameter, CAS = circumferential artery strain, ODI = oxygen desaturation index, SaO2 = arterial oxygen saturation.

The systolic peak global CAS was significantly inversely correlated with the β value (r = −0.61, *P* < .05), the age (r = −0.34, *P* < .05), the SaO_2_ (r = 0.63, *P* < .01), and the oxygen desaturation index (ODI) (r = −0.67, *P* < .01) (Table 4, Fig. 3).

**Table 4:**
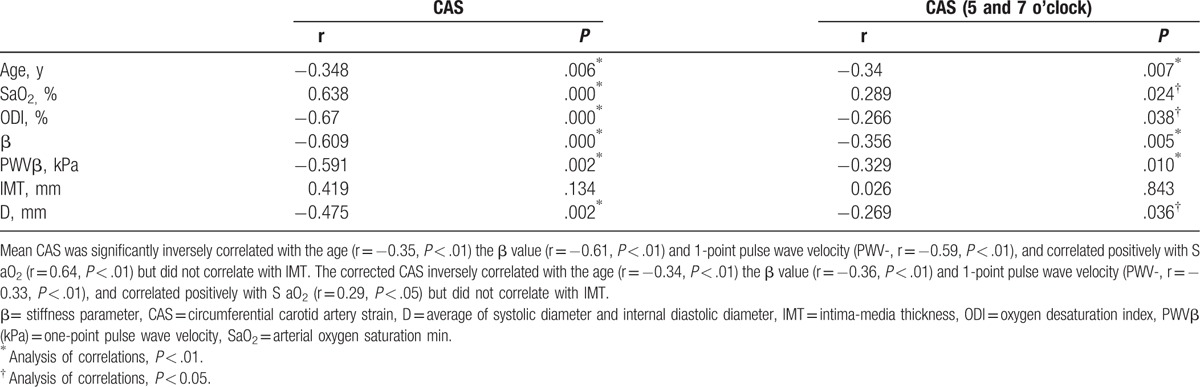
Correlations between mean CAS and corrected CAS and clinical variables.

**Figure 4:**
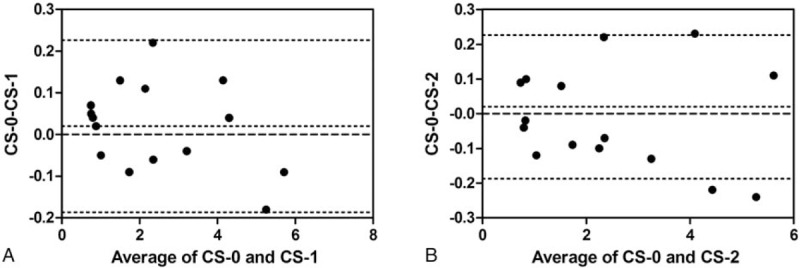
The horizontal solid line indicates the mean difference between the 2 observers, and the dashed lines indicate the 95% limits of agreement (mean difference ± 1.96 times the SD of the difference). Interobserver and intraobserver coefficients of variation of CAS were (−0.17 to 0.19) mm, (−0.23 to 0.19) mm. A, Interobserver coefficient of variation of CAS. B, Intraobserver coefficient of variation of CAS. CAS = circumferential artery strain.

The limits of agreement of the CAS between and within observers were listed in Fig. 4. The limits of agreement of the CAS between and within observers were (−0.17 to 0.19) and (−0.23 to 0.19). The range of the difference could be tolerated. Interobserver and intraobserver variability of CAS was 5.01% and 5.45% respectively.

To further explore the possible factors affecting the systolic peak global CAS in OSAHS patients, the multiple linear regression analyses was performed. Consequently, the age and oxygen desaturation index accounted for 19.9% of the variation of the systolic peak global CAS, when all variables that were significantly correlated with the systolic peak global CAS were included as independent variables (Table 5). The prevalence of OSAHS was positively correlated with age. The higher the age, the prevalence of OSAHS was also increased. The prevalence of OSAHS was also positively correlated with ODI. The higher the ODI, the prevalence of OSAHS was also increased.

**Table 5:**
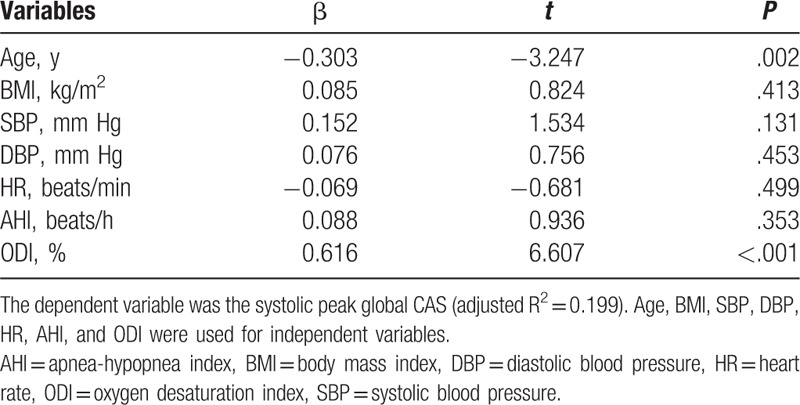
Stepwise multiple linear regression analyses of factors affecting the systolic peak global CAS in OSAHS patients.

## 4 Discussion

OSAHS is a relatively common sleep disorder characterized by recurrent nocturnal apnea due to partial or complete upper airway collapses.^[6]^ OSAHS causes a series of severe sequelae, including intermittent hypoxia exposure,^[7]^ chronic daytime sleepiness, and decreased quality of life.^[8]^ Many evidences have shown recently that OSAHS is an independent risk factor for the development of cardiovascular and cerebrovascular events.^[9]^ Intermittent hypoxia associated with OSAHS may play the vital role in the coagulation function of arterial blood vessels and the development of atherosclerosis.^[10]^ The accelerated development of atherosclerosis can explain the connection between OSAHS and CVD.^[11]^ In summary, OSAHS is significantly associated with cardiovascular and cerebrovascular events morbidity and mortality.^[12]^

In this study, an increase in arterial stiffness, the β and PWVβ in severe group were higher than those in the control group and the mild group, the differences were statistically significant. The main mechanism of the decreased elasticity and increased stiffness of the carotid artery may be as follows: the OSAHS patients have severely impaired endothelial function and that exposure to intermittent hypoxia may contribute to endothelial dysfunction and vascular remodeling.^[13]^ Previous studies indicate that intermittent hypoxia causes inflammation and oxidative stress by stimulating vascular superoxide production and limits bioavailability of nitric oxide.^[14]^ Additionally, both OSAHS and CVD have been linked to changed diurnal profiles of enormous agents authoritatively influencing vascular tone and endothelial cell migration, proliferation.^[15]^ Intermittent hypoxia associated with OSAHS significantly influences the cardiovascular system through actions on sympathetic nervous system^[16]^ and endothelial function.

Many studies had shown that decreased arterial elasticity was not only one of the early signs of atherosclerosis, but also an independent risk factor and predictor of cardiovascular events morbidity and mortality.^[17]^ Our objective was to use the new measurement technique to detect blood vessel aging in LCCA and to clarify differences in arterial mechanical performance.^[18]^ The previous development of 2D-STI provides the methods of evaluating left ventricular tissue function.^[10]^ 2D-STI is a relatively new method of angle-independent quantification of left ventricular tissue strain, the angle of rotation, and mechanical parameters of myocardium.^[19]^ Analysis of arterial wall mechanics using 2D-STI become available not only to acquire radial and longitudinal displacement but also to calculate circumferential strain.^[20]^ As arteries age, smooth muscle cells decrease and degenerate in number through apoptosis of cells, and the elastin layer becomes fragmented.^[21]^ To increase with normal aging, these processes lead to increased arterial stiffness. Bjallmark et al^[22]^ used the speckle-tracking analysis of the only right carotid artery strain in their studies and checked for a smaller population specimen. They did not report on the interobserver reproducibility of their measurements. Our study was designed to measure the reliability and reproducibility of CAS by 2D-STI in the common carotid artery. Bland–Altman analysis diagram showed each point was scattered in the 95% confidence interval between the interobserver and intraobserver. The CAS and β had good interdependency, which showed that the results of these 2 indications were in good constancy. 2D-STI could be used as a new method to evaluate carotid artery elasticity and vascular remodeling.

The systolic peak global CAS in the moderate and severe groups were lower than in the mild group. The segment CAS values between 5 and 7 o’clock in the moderate and severe groups were lower than in the mild group. In the moderate and severe groups, inflammation is recognized as playing a crucial role in the atherosclerotic disease. A battery of circulating levels of inflammatory molecules have been measured, with the purpose of investigating inflammation and calculating vascular injury risk, such as C-reactive protein (CRP),^[23]^ adhesion molecules,^[24]^ cytokines.^[25]^ Therefore, evaluation of circulating biological tag of inflammation, including cytokines and adhesion molecules, has become recognized as a useful instrument for identifying patients at highest risk for cardiovascular events.^[26]^ The chemokine plays an important role in atherogenesis by enhancing oxidative stress, and mediating adhesion of monocytes to the arterial endothelium.^[27]^ The chemokine levels are increased in OSAHS patients in comparison with dominates blended for alternating quantities such as body mass index and age.

The global CAS was significantly inversely interrelated with β and PWVβ. In our study, CAS value is off-line semiautomatic measurement^[28]^ using acoustic-tracking software and has high reliability and repeatability. Compared with β and PWVβ, the CAS measurement reliability and repeatability may be better. In addition, the 2D-STI is an echocardiographic method that uses B-mode images for tracking of acoustic- speckles analysis.^[29]^ It has the advantage of being angle-independent, and to be less interfered with minor lobes, reverberations^[30]^ and leave off artifacts. Through stepwise multiple linear regression analysis, age and ODI were the important variable quantities that determined the global CAS. These mechanisms are considered to strengthen the association of OSAHS with investigate inflammation and calculate vascular injury risk. For this reason, the ODI can better explain the CAS and reduce OSAHS order of severity compared with AHI and SaO2.

Currently, an ultrasound method of the carotid arteries is obligatory in all cases with cardiovascular and cerebrovascular events. A more detailed examination that includes CAS measurements in patients with cardiovascular risk may therefore be of value when testing the risk of arterial aging.

There are a few limitations related to this study. First, the OSAHS sample size was small, the radial and longitudinal strain should also be tested. Second, the effects of higher frame rates on repeatability of strain measurements are uncertain.

## 5 Conclusions

2D-STI is a feasible and reproducible measurement for the assessment of carotid arterial elasticity in patients with OSAHS. This technique may be more sensitive to changes from abnormal arterial function than previous techniques based on luminal measurements.
